# Recurrent choriocarcinoma complicated with leprosy during chemotherapy: A case report and literature review

**DOI:** 10.1097/MD.0000000000034548

**Published:** 2023-08-11

**Authors:** Shiqi Hu, Xiaojuan Lin, Rutie Yin, Wei Wang, Qingli Li

**Affiliations:** a Department of Gynecology and Obstetrics, Development and Related Diseases of Women and Children Key Laboratory of Sichuan Province, Key Laboratory of Birth Defects and Related Diseases of Women and Children, Ministry of Education, West China Second University Hospital, Sichuan University, Chengdu, P. R. China; b Department of Pathology, Development and Related Diseases of Women and Children Key Laboratory of Sichuan Province, Key Laboratory of Birth Defects and Related Diseases of Women and Children, Ministry of Education, West China Second University Hospital, Sichuan University, Chengdu, P. R. China.

**Keywords:** chemotherapy, choriocarcinoma, gestational trophoblastic neoplasia, immunotherapy, infection, leprosy

## Abstract

The global prevalence of leprosy has decreased substantially, and cases of leprosy infection are extremely rare in China. In this report, we present a case of recurrent choriocarcinoma complicated by leprosy infection during chemotherapy.

**Patient concerns:**

A 24-year-old Chinese woman (gravida 3, para 2) presented to a local hospital with vaginal bleeding. Her medical history included a previous diagnosis of hydatidiform mole.

**Diagnoses, Interventions and Outcomes:**

The patient was diagnosed with choriocarcinoma and received chemotherapy in 6 cycles. Shortly after the initial treatment was completed, the disease recurred twice with resistance to multiple chemotherapeutic agents. In her second recurrence of choriocarcinoma, she was diagnosed with leprosy with many cutaneous nodules throughout her entire body. The patient was administered chemical treatment for leprosy with the multidrug therapy regimen after being diagnosed. To prevent exacerbating the infection, no immunotherapy was utilized to treat cancer, and the infection was well-controlled at the conclusion of anticancer therapy.

**Lessons:**

Because of immunological reduction, cancer patients are susceptible to a variety of infections. For patients with cancer, prevention and early detection of rare infectious diseases should receive special attention. Immunotherapy must be used with caution when treating patients with cancer and infections.

## 1. Introduction

Leprosy is caused by Mycobacterium leprae. This chronic infectious disease mostly affects the skin and peripheral nerves, and in severe cases can result in disability.^[1]^ In 1982, the World Health Organization introduced multidrug therapy (MDT) for leprosy.^[2]^ In recent decades, the prevalence of leprosy has decreased significantly around the world as a result of improved treatment and advancements in medical care. By 2000, the prevalence of leprosy had dropped to < 1 case per 10,000 people worldwide.^[3]^ Nonetheless, the elimination of leprosy had not yet been accomplished, and new cases continue to emerge.

Gestational trophoblastic neoplasia (GTN) is caused by aberrant proliferation of placental trophoblastic cells. Although GTN is highly responsive to chemotherapy, drug resistance and relapse remain a challenge for individual patients. Choriocarcinoma, a subtype of GTN, is highly aggressive and characterized by early distant metastases.

There have been no reported cases of leprosy among patients diagnosed with GTN or choriocarcinoma. This case study initially described a patient with recurrent choriocarcinoma who developed leprosy following chemotherapy. In addition, we conducted a literature review and discussed the relationship between leprosy and choriocarcinoma, as well as the treatment strategy.

## 2. Case presentation

A 24-year-old Chinese woman with gravida 3 and parity 2 presented to a primary hospital located nearby because of vaginal bleeding 2 months after her second full-term delivery in July 2020. Her medical history included a previous diagnosis of hydatidiform mole in 2016 and a subsequent normal delivery in 2019. Physical examination revealed a 2 cm nodule on the vaginal wall. Histopathological examination of the vaginal mass revealed the presence of GTN. Additionally, immunohistochemical analysis revealed strong positivity for human chorionic gonadotropin (hCG) and cytokeratin 7, focal positivity for spalt-like transcription factor 4, isolated positivity for P63, negativity for human placental lactogen and leukocyte common antigen, and Ki-67 positivity index exceeding 80%. Choriocarcinoma was diagnosed based on these findings. The patient’s serum hCG level was 264,000 mIU/mL, and a computed tomography (CT) scan of the chest showed multiple metastatic nodules in both lungs. As a result, the patient was classified as FIGO stage III with a risk factor score of 7. Six subsequent cycles of BEP chemotherapy (bleomycin, etoposide, and platinum) were administered at the local hospital, and her hCG levels normalized after the 4th cycle.

The patient’s serum hCG level increased to 1435.10 mIU/mL 1 month after the completion of the initial chemotherapy. In March 2021, the patient sought medical treatment at our hospital. Contrast-enhanced CT scan revealed the presence of a mass with soft tissue density in the uterine cavity, which exhibited inhomogeneous enhancement and measured 2.7 cm × 2.2 cm. Furthermore, 2 nodules with dimensions of 1.6 cm × 1.4 cm and 1.4 cm × 1.4 cm were detected in the right lung, which were indicative of metastatic spread. Nevertheless, the results of brain magnetic resonance imaging indicated the absence of any irregularities. The patient was diagnosed with the first recurrence of choriocarcinoma at stage III with a risk score of 11. The patient underwent treatment with the EMA-CO chemotherapy regimen, which included etoposide, methotrexate, actinomycin-D, cyclophosphamide, and vincristine. The patient’s serum hCG level was negative after 3 cycles of EMA-CO treatment. Following this, 4 cycles of consolidation therapy were sequentially administered, and the patient’s serum hCG level remained below 2 mIU/mL. Upon completion of the chemical treatment, a CT scan was performed, which revealed a reduction in the size of the tumor masses located in the uterine (2.3 cm × 2.0 cm) and right lung (0.7 cm × 0.4 cm and 0.5 cm × 0.2 cm) regions, as compared to the previous examination.

**Figure 1.**
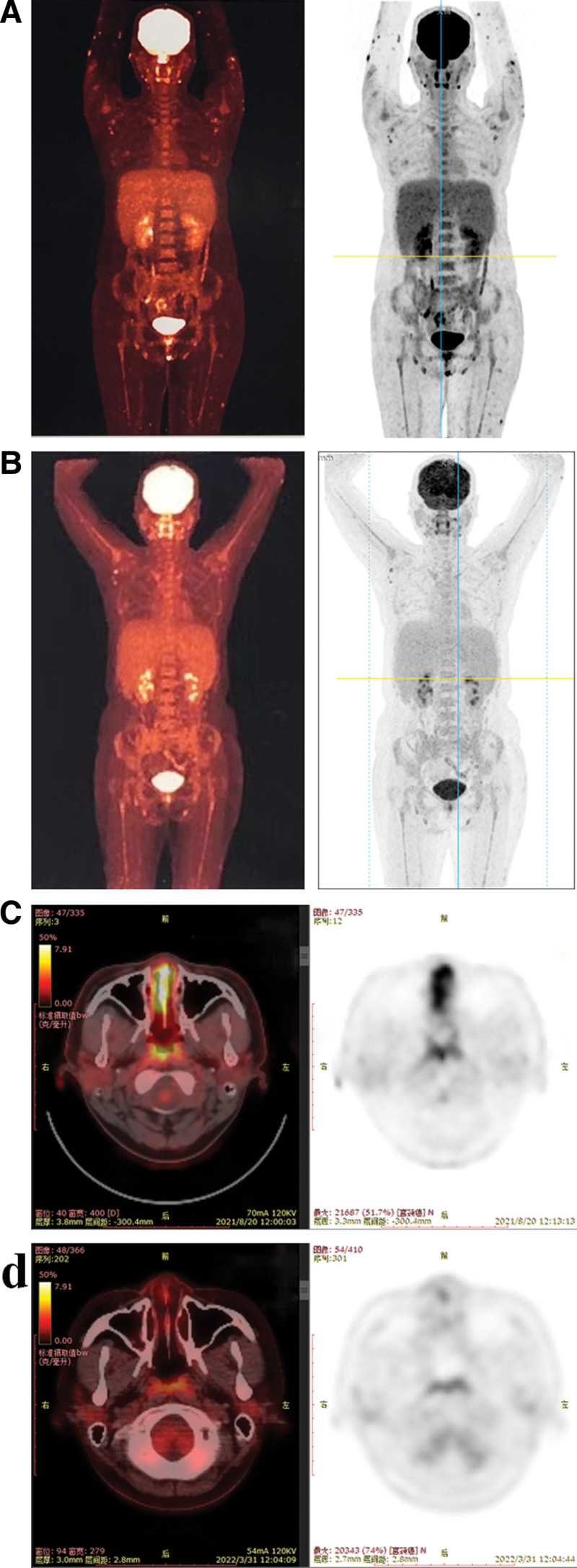
Positron emission tomography computed tomography (PET-CT) images. (A) Multiple cutaneous nodules in the whole body with increased glucose metabolism before leprosy therapy. (B) The cutaneous lesions after leprosy therapy. (C) Thickened nasal mucosa with increased glucose metabolism before leprosy therapy. (D) The lesions of nasal mucosa after leprosy therapy.

**Figure 2.**
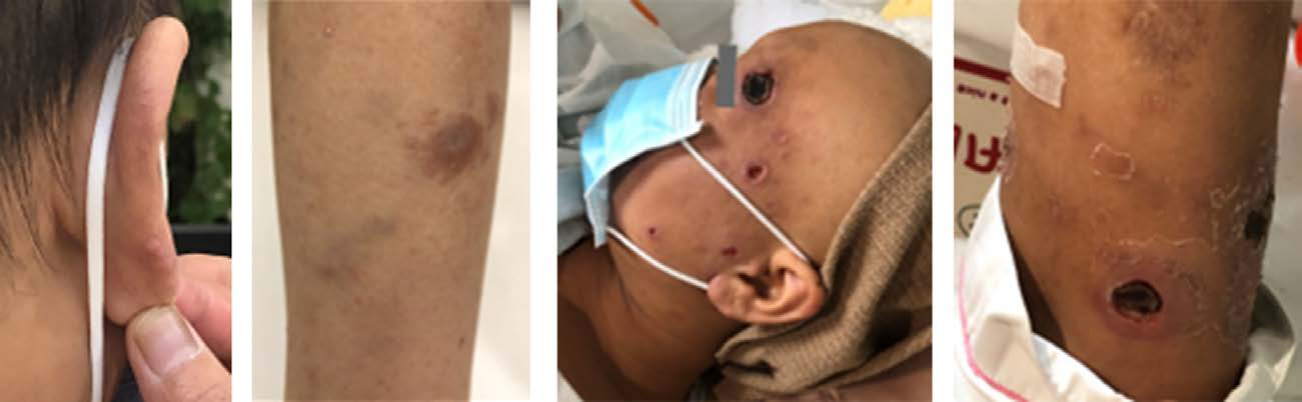
The cutaneous lesions of leprosy in the patient’s arms, face and ears.

**Figure 3.**
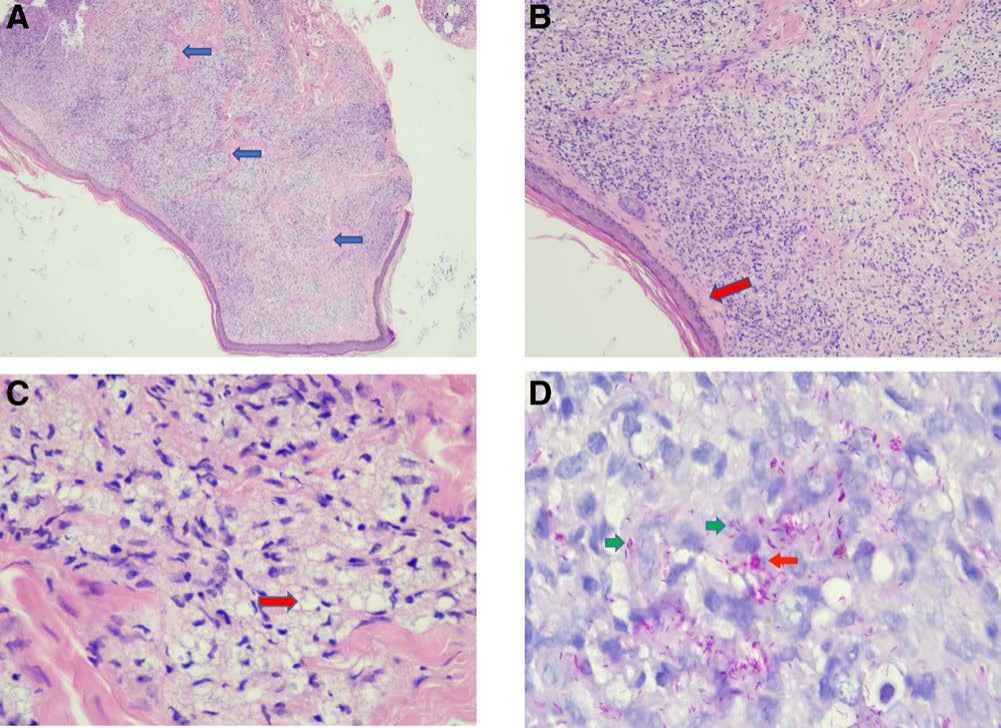
Histopathological examination. (A) Lepra granuloma in dermis (×40). (B) A non-infiltrating zone between the epidermis and dermis (×100). (C) A large number of foam cells (×400). (D) Abundant acid-fast positive bacilli, formed globus leprosus (×1000).

In August 2021, approximately 2 months post-therapy, the patient’s serum hCG levels exhibited a resurgence of 3266.8 mIU/mL. Positron emission tomography computed tomography showed several abnormalities (Fig. 1A and C), including a cancerous growth in the uterine cavity, numerous metastatic nodules in both lungs, a metastatic lesion near the left lateral ventricle, and thickened nasal mucosa, along with multiple cutaneous nodules throughout the body with increased glucose metabolism. The patient manifested hyperpigmentation of the skin and several brown nodules with varying diameters between 0.3 cm to 1.0 cm on the arms, legs, and face during the physical examination (Fig. 2). The diagnosis of a second recurrence of choriocarcinoma (stage IV: 15) was made based on the presence of brain metastasis, placing the patient in the ultra-high-risk group for GTN. Skin biopsy was performed on the cutaneous nodes to confirm the diagnosis. Histopathological analysis indicated the existence of circular, polygonal, or spindle-shaped cells proliferating in the dermis or subcutis with a profusion of acid-fast positive bacilli, suggesting mycobacterial infection. Histopathological examination also showed the presence of a large number of foam cell granulomas in the dermis, which contained multiple bacterial clusters (Fig. 3). Additionally, a non-infiltrating zone was observed between the epidermis and dermis (Fig. 3B). Moreover, acid-fast staining of the skin smears obtained from 6 cutaneous lesions resulted in positive outcomes. These findings confirmed a diagnosis of lepromatous leprosy. The patient denied any past record of contact with people diagnosed with leprosy. She reported the emergence of painless nodules in her earlobes and arms during her last pregnancy in 2020. Subsequently, the size and quantity of nodules has increased in recent years. The World Health Organization MDT regimen, consisting of rifampicin, clofazimine, and dapsone, was used to treat the infection.

Concurrently, the patient continued to undergo chemotherapy treatment for the relapse of choriocarcinoma utilizing the EP (etoposide and platinum)/EMA (etoposide, methotrexate, actinomycin-D) therapeutic regimen. After 4 cycles of EP/EMA, the patient’s serum hCG level exhibited a rebound from 35.1 mIU/mL to 91.0 mIU/mL. Following a multidisciplinary discussion, the chemotherapy regimen for the 5th cycle was changed to FAEV (floxuridine, actinomycin-D, etoposide, and vincristine), and the treatment was continued for an additional 8 cycles, comprising 5 consolidation cycles. A follow-up positron emission tomography computed tomography was conducted and the results (Fig. 1B and D) indicated the following: Absence of residual lesions in the uterus; Significant reduction in the size of metastatic nodules in the right lung; Significant decrease in the size of the left lateral paraventricular lesion compared to previous results; Noticeable reduction in the size, quantity, and extent of glucose metabolism in previously identified lesions located in the nasal mucosa and skin. Radiotherapy was administered 3 times after chemotherapy to resolve the residual brain metastasis. Within 2 months of the completion of chemotherapy, the patient’s serum hCG level was monitored and found to be normal.

## 3. Discussion

The patient was diagnosed with leprosy during the second choriocarcinoma recurrence. Individuals who develop malignant tumors, particularly those receiving chemotherapy, may experience an elevated susceptibility to leprosy due to immune suppression. The clinical symptoms and histological changes in patients with leprosy are determined by their immune status.^[1]^ Because of the patient’s immune deficiency, she presented with lepromatous leprosy, a form of the disease characterized by the presence of numerous bacteria in lesions and a high level of infectivity. The association between cancer and leprosy remains uncertain, and to date, no relevant studies or reports have been published.

Tuberculosis (TB) and leprosy are both caused by mycobacterial infections and exhibit similarities in their pathogenesis and management. Numerous studies have investigated the risk of TB infection in cancer patients. Existing research has revealed that patients with malignant tumors have a higher risk of developing TB.^[4–6]^ A total of 16,487 cancer patients and 65,948 controls were recruited for a nationwide population-based retrospective cohort study to assess the prevalence of active TB. They discovered that patients with cancer had a higher incidence of TB (339 per 100,000 person-years) than controls (202 per 100,000 person-years). Furthermore, malignancies of the aerodigestive tract, lung, and hematologic system considerably elevate the risk.^[6]^ Although some studies have suggested that patients with hematologic malignancies are at an increased risk of developing TB,^[7–9]^ a retrospective cohort analysis found that people with solid-organ malignancies were also more likely to develop TB than people who did not have cancer.^[4]^ The suppression of the immune system, either as a result of cancer or antitumor chemotherapy, may heighten susceptibility to TB infection. According to a study, 90.9% of patients diagnosed with active TB subsequent to cancer diagnosis had a prior history of healed TB.^[4]^ On the basis of this result, it was more probable that the infection was caused by latent TB reactivation than by a new TB infection. On the other hand, a number of studies have revealed that TB patients have an increased risk of cancer, including some types of extrapulmonary cancer.^[10–12]^ Cancer may develop as a result of chronic inflammation at local and systemic levels of TB infection, and populations with TB infection and certain types of cancer may share some common risk factors.

Although numerous studies have been conducted on cancer and TB, there is a dearth of research on leprosy. Based on the homogeneity of the pathogens involved in TB and leprosy, we hypothesized that an interaction might exist between leprosy infection and choriocarcinoma in this case. Upon reviewing the patient’s medical records, it was found that she presented with several small, painless skin nodules on her hands and feet during her previous pregnancy. However, no definitive diagnosis was made at that time. It is likely that these nodules were early skin lesions of leprosy that subsequently progressed to lepromatous leprosy during the course of antitumor chemotherapy. Nevertheless, the potential impact of leprosy infection on choriocarcinoma progression remains unclear.

The patient initially received treatment at a local hospital where a nonstandard BEP chemotherapy regimen was administered. After being diagnosed with the first recurrence of choriocarcinoma, the patient sought treatment at our hospital and was administered the standard first-line EMA-CO chemotherapy regimen. However, shortly after completing chemotherapy, the patient experienced a second recurrence, which was characterized by an ultra-high-risk GTN according to the FIGO staging and scoring. In addition, resistance to medication has been observed during chemotherapy. As 1 type of immune checkpoint inhibitors (ICIs), programmed cell death protein 1 (PD-1)/programmed death ligand 1 (PD-L1) inhibitors showed some therapeutic benefit for ultra-high-risk GTN patients with multiagent chemotherapy resistance.^[13–15]^ Several studies have demonstrated a high level of PD-L1 expression in GTN,^[16–18]^ suggesting that PD-1/PD-L1 inhibitors may be an effective therapeutic strategy. However, when treating cancer patients who also suffer from infections, the use of immunotherapy should be approached with caution. Recently, several cases of TB infection activation in cancer patients receiving ICIs treatment have been reported,^[19–23]^ According to a meta-analysis, the incidence of TB was 35 times higher in PD-1/PD-L1 blockade-treated patients than in the general population.^[24]^ Meanwhile, another observational study revealed that among patients with cancer, the incidence of TB in the group exposed to ICIs was 675.8 per 100,000 person-years (95% confidence interval: 412.8–1043.8), while in the non-exposure group, it was 599.1 per 100,000 person-years (95% confidence interval: 560.5–639.6).^[25]^ This result indicated that cancer patients had a higher incidence of TB infection than the general population, but exposure to ICIs did not differ significantly. The results of previous animal experiments indicated that mice deficient in PD-1 exhibited a significantly heightened susceptibility to TB infection, and their survival time was notably reduced in comparison to wild-type mice. Researchers have found that PD-1-deficient mice develop large necrotic lesions and a higher mycobacterial burden in their lungs.^[26]^ Although it is unclear what role the PD-1/PD-L1 signal pathway plays in TB infection, murine studies have shown that T cells lacking PD-1 mediated inhibition may promote rather than control the infection of TB.^[27,28]^ Based on the aforementioned reports, the association between immunotherapy and TB infection remains unclear. However, suppression of the PD-1/PD-L1 pathway may promote the development of TB.

In 2021, the Spanish Melanoma Group panel developed cancer immunotherapy guidelines for special populations facing challenging conditions, including patients with TB infection. The guidelines recommended that if an active TB infection was confirmed, TB therapy should be administered prior to initiating immunotherapy.^[29]^ Furthermore, a reported case of nodular melanoma was found to have arisen from a trophic ulceration in a patient afflicted with leprosy.^[30]^ After surgery, the patient received IFNα-2b immunotherapy, with no major side effects recorded. Unfortunately, this therapy is not suitable for the treatment of GTN. In our case, the patient had an active infection, which was diagnosed as lepromatous leprosy, and was treated with the MDT regimen for leprosy soon after diagnosis. Owing to the possibility of infection exacerbation, we did not add a PD-1/PD-L1 inhibitor to her treatment regimen. Finally, leprosy infection was effectively controlled at the conclusion of antitumor chemotherapy.

## 4. Conclusion

This is the first reported case of leprosy infection in a patient with a recurrent choriocarcinoma. The second recurrence of choriocarcinoma was accompanied by the detection of multiple cutaneous nodules throughout the body, ultimately resulting in a diagnosis of leprosy. As a result of immunological reduction, patients with cancer are susceptible to a variety of infections. Particular attention should be paid to the prevention and early detection of rare infectious diseases. When it comes to treating cancer patients with infections, caution must be exercised because cancer immunotherapy has the potential to modify the immunological status, which could activate or exacerbate infections. Therefore, immunotherapy should be used with care in patients with active infections, and chemoprophylaxis can be considered for high-risk patients before starting immunotherapy.

## Author contributions

**Conceptualization:** Shiqi Hu, Xiaojuan Lin, Rutie Yin, Qingli Li.

**Data curation:** Shiqi Hu, Wei Wang, Qingli Li.

**Funding acquisition:** Xiaojuan Lin, Qingli Li.

**Supervision:** Qingli Li.

**Visualization:** Shiqi Hu, Wei Wang.

**Writing – original draft:** Shiqi Hu, Qingli Li.

**Writing – review & editing:** Xiaojuan Lin, Rutie Yin.
